# Impact of Diet and Maternal Obesity on Human Milk Hyaluronan

**DOI:** 10.3390/nu17223560

**Published:** 2025-11-14

**Authors:** Christopher Hoover, Karni S. Moshal, Jeffrey V. Eckert, Adam P. Wilson, Kathryn Y. Burge, David A. Fields, Hala Chaaban

**Affiliations:** 1 Department of Pediatrics, The University of Oklahoma Health Campus, Oklahoma City, OK 73104, USA; christopher-hoover@ou.edu (C.H.); david-fields@ou.edu (D.A.F.); 2 Section of Neonatal-Perinatal Medicine, Department of Pediatrics, The University of Oklahoma Health Campus, Oklahoma City, OK 73104, USA; karni-moshal@ou.edu (K.S.M.); jeffrey-eckert@ou.edu (J.V.E.); adam-wilson@ou.edu (A.P.W.); kathryn-burge@ou.edu (K.Y.B.)

**Keywords:** human milk, breast milk, human milk bioactives, hyaluronan, maternal obesity, dietary intake

## Abstract

**Background:** Human milk hyaluronan (HA), a glycosaminoglycan with barrier-protective and immunomodulatory functions, may be influenced by maternal characteristics. The effects of maternal obesity and acute dietary intake on milk HA concentrations remain unclear. **Methods:** This secondary analysis included 35 lactating mothers (*n* = 19 normal weight [NW], *n* = 16 obese [OB]) at 6 weeks postpartum who participated in two separate, but standardized, protocols: (1) Study One, which consisted of hourly milk collections for six hours following a standardized high-fat meal with a sugar-sweetened beverage beginning at 6:00 am, and (2) Study Two, which consisted of daily morning milk collections for seven consecutive days to assess temporal stability (Monday-Sunday). HA concentrations were quantified by an ELISA and analyzed using a mixed-effects and repeated-measures ANOVA. **Results:** In Study One, postprandial HA concentrations remained stable with no effect of time, BMI, or time × BMI interaction (*p* > 0.05). In Study Two, HA did not vary significantly by day (*p* = 0.082) but was higher in OB versus NW mothers (151.9 ± 18.7 vs. 96.5 ± 12.4 ng/mL; *p* = 0.0396), with the largest difference observed on Day 1 (*p* = 0.0117). Mean HA values trended upward later in the week (Day 6 and 7), suggesting potential influences of habitual dietary intake or weekend energy patterns. **Conclusions:** Milk HA concentrations were not altered by acute dietary intake but were consistently higher across multiple days in mothers with obesity. These results indicate that milk HA varies with maternal metabolic status and may also be influenced by habitual dietary patterns, including fluctuations between weekday and weekend intake.

## 1. Introduction

Human milk (HM) is a complex biological fluid that delivers both essential nutrients and diverse bioactive molecules supporting infant growth, immune development, and metabolic regulation [1]. In addition to macronutrients and micronutrients, HM contains hormones, cytokines, oligosaccharides, glycoproteins, and a diverse microbiota, all of which shape early development and long-term health outcomes [2]. Both maternal metabolic status and dietary intake have been shown to influence HM composition [3,4]. Obesity has been associated with higher concentrations of leptin, insulin, and pro-inflammatory cytokines, as well as changes in lipid profiles and HM oligosaccharides (HMOs) [5,6,7,8,9,10,11,12]. Similarly, maternal diet can modulate milk bioactives, including lipids and certain immunomodulatory proteins [13,14]. These alterations have been linked to differences in infant adiposity, immune development, and infant gut microbiome composition [15,16].

Hyaluronan (HA), a non-sulfated glycosaminoglycan, is increasingly recognized as a key bioactive constituent of HM [17,18]. HA supports epithelial barrier integrity, modulates inflammatory signaling, and shapes microbial colonization of the intestine [19,20,21]. HA’s concentration peaks in colostrum and early milk within the first two weeks of lactation and declines with advancing lactation [22]. HM-derived HA and low-molecular-weight HA of ~35 kDa (HA35) have been shown to protect against intestinal inflammation and injury in multiple in vivo models, including experimental necrotizing enterocolitis (NEC) [17,18]. Collectively, these findings suggest that HA may contribute to regulation of infant gut and immune health.

Despite its biological relevance, the determinants of milk HA concentrations remain poorly understood. Obesity is characterized by chronic low-grade inflammation, insulin resistance, and altered glucose metabolism, factors known to influence HA synthesis and degradation [23,24]. Whether these obesity-associated metabolic disturbances or maternal dietary factors affect HA concentrations in HM remains unknown.

To address these gaps, we conducted a secondary exploratory analysis of stored milk samples from two study protocols: (1) Study One, which involved serial postprandial sampling following a standardized high-fat meal and sugar-sweetened beverage to examine acute dietary effects, and Study Two, which involved daily pre-prandial sampling across seven consecutive days (7:00 am) to assess temporal stability and the influence of maternal BMI on HM HA levels. The exploratory work was designed to inform whether maternal obesity and diet should be incorporated as key covariates for future prospective studies of HA in human milk.

## 2. Materials and Methods

This study is a secondary analysis combining data from two prospective cohort studies conducted at the University of Oklahoma Health Sciences Campus (OUHSC) and the University of Minnesota [25,26]. Both parent studies obtained written informed consent at the time of enrollment. The current analysis, which involved measurement of HM HA in stored samples and use of de-identified clinical data, was approved by the Institutional Review Board with a waiver of additional consent.

Eligible participants were women with a pre-pregnancy BMI of 18.5–40 kg/m^2^, aged 21–45 years at delivery, who experienced an uncomplicated term birth with a maternal hospital stay <3 days, and delivered a healthy singleton infant weighing 2500–4500 g. Mothers were enrolled only if intending to exclusively breastfeed for at least 3 months. Exclusion criteria included alcohol, tobacco, or recreational drug use during pregnancy or lactation; pre-existing diabetes; or delivery of an infant with congenital anomalies affecting feeding or growth. For this analysis, HA was quantified in available stored milk samples from 35 lactating women at OUHSC who had participated in the two parent protocols. Demographic and clinical data were obtained primarily through medical record abstraction.

At 6 weeks postpartum, participants completed two standardized milk collection protocols using a hospital-grade electric breast pump (Symphony PLUS, Medela, McHenry, IL, USA), and a full breast expression was obtained at each time point. Samples were frozen immediately at −20 °C and transferred to −80 °C for long-term storage. Study One (acute dietary challenge) included all 35 mothers. After an overnight fast, a baseline sample was collected at 6:00 am, followed by consumption of a standardized breakfast and a 20-oz sugar-sweetened soft drink; milk was then collected hourly for six hours (6:00–12:00) (*n* = 35). Study Two (7-day temporal stability) was completed by a subset of participants who consented to the extended sampling schedule. 18 of the original 35 mothers provided milk samples at 7:00 am on seven consecutive days (Monday–Sunday). For both protocols, participants were instructed to pump a complete breast expression at each collection, ensuring inclusion of fore-, mid-, and hind-milk. Samples were obtained from the right breast unless milk was only available from the left. Following collection, participants immediately froze each sample according to standardized handling procedures. Trained study staff retrieved the frozen samples within two days, transported them on ice to OUHSC, and immediately stored them at −80 °C. HA concentrations were measured in thawed aliquots by enzyme-linked immunosorbent assay (ELISA; Echelon Biosciences, K-1200, Salt Lake City, UT, USA) according to manufacturer instructions and previously described procedures, and are reported as ng/mL [22].

Descriptive statistics are presented as mean ± SEM for continuous variables and *n* (%) for categorical variables. Normality and homoscedasticity were assessed with Shapiro–Wilk and Levene’s tests. Baseline group differences used independent-samples *t*-tests (continuous) and Mantel–Haenszel χ^2^ tests (categorical), and effect sizes for all pairwise comparisons were calculated using Cohen’s *d*. For Study One, postprandial trajectories were analyzed using mixed-effects models (restricted maximum likelihood) with fixed effects for time, BMI group (normal weight [NW] ≤ 25 kg/m^2^; obese [OB] ≥ 30 kg/m^2^), and the time × BMI interaction, and a random intercept for subject to account for within-participant correlation and intermittent missingness. Effect sizes for fixed effects (main effects and interactions) were summarized using partial η^2^. Tukey’s post hoc tests compared baseline with each postprandial time point and contrasted BMI groups at each time. For Study Two, mothers were stratified by pre-pregnancy BMI (NW vs. OB), and a two-way repeated-measures ANOVA tested main effects of BMI group, time, and the BMI × time interaction, with partial η^2^ reported for all effects. Tukey’s post hoc tests were used for pairwise comparisons (between days and between BMI groups). Two-sided *p* < 0.05 was considered statistically significant.

## 3. Results

### 3.1. Participant Characteristics

A total of 35 lactating mothers were included in this sub-analysis, stratified by pre-pregnancy BMI. As expected, mothers in the OB group had higher pre-pregnancy weight (86.5 ± 19.8 vs. 59.3 ± 5.9 kg, *p* < 0.0001), lower height (157 ± 9.8 vs. 163 ± 6.3 cm, *p* = 0.03), and higher BMI (34.3 ± 4.6 vs. 22.1 ± 1.4 kg/m^2^, *p* < 0.0001) compared with NW mothers. Maternal age and parity did not differ significantly (Table 1).

### 3.2. Effect of Acute Maternal Diet on HM HA Concentrations

**Figure 1:**
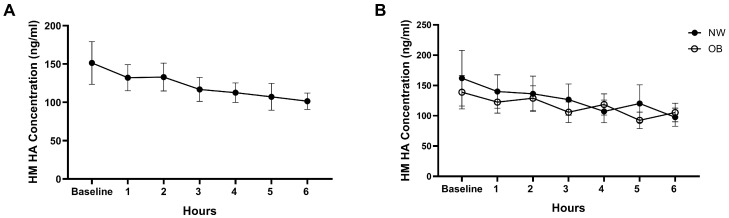
Milk hyaluronan (HA) concentrations following a dietary challenge. (**A**) Hourly HA concentrations measured over 6 h after a high-fat meal with a sugar sweetened beverage (*n* = 35). A modest decline was observed but did not reach significance (*p* = 0.058; partial η^2^ = 0.07). (**B**) HA concentrations stratified by maternal pre-pregnancy BMI (NW, *n* = 19; OB, *n* = 16). No significant effect of BMI group (*p* = 0.77; Cohen’s *d* = 0.15), time (*p* = 0.066; partial η^2^ = 0.07), or BMI × time interaction (*p* = 0.62; partial η^2^ = 0.02) was detected. Data are shown as mean ± SEM.

Across the cohort, HA concentrations showed a downward trend from baseline (151.3 ng/mL) to 6 h post-meal (101.4 ng/mL; *p* = 0.058, Cohen’s *d* = 0.40), indicating a small-to-moderate effect size. Mixed-effects modeling indicated that time accounted for a modest proportion of variance (partial η^2^ = 0.07), whereas variability accounted for the majority of variance (*p* < 0.0001; Figure 1A).

When stratified by BMI, mean HA concentrations were comparable between NW and OB groups (124.9 vs. 116.1 ng/mL, *p* = 0.77, Cohen’s *d* = 0.15). Neither the main effect of time (*p* = 0.066; partial η^2^ = 0.07) nor the BMI × time interaction (*p* = 0.62; partial η^2^ = 0.02) reached statistical significance. No group differences at individual time points were detected. Within-group analyses similarly showed no consistent postprandial pattern in either BMI group, indicating that acute dietary intake did not significantly influence HA concentrations (Figure 1B).

### 3.3. Temporal Stability of HM HA Concentrations

Eighteen mothers completed the 7-day temporal stability protocol. Maternal age was comparable between groups, and parity did not differ significantly. As expected, mothers in the OB group had higher pre-pregnancy weight (82.7 ± 8.9 vs. 57.1 ± 4.83 kg, *p* < 0.001) and BMI (33.0 ± 1.88 vs. 22.1 ± 1.56 kg/m^2^, *p* < 0.001), whereas pre-pregnancy height did not differ. Full demographic details for this subset are provided in Supplemental Table S1.

**Figure 2:**
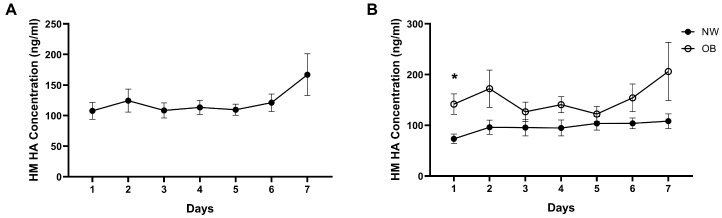
Milk hyaluronan (HA) concentrations across seven consecutive days. (**A**) Daily HA concentrations in all mothers combined (*n* = 18). No significant effect of time was observed *(p =* 0.082; partial η^2^ = 0.11). (**B**) HA concentrations stratified by maternal pre-pregnancy BMI (NW, *n* = 10; OB, *n* = 8). Mothers in the OB group exhibited significantly higher HA concentrations compared with NW mothers (*p* = 0.028; partial η^2^ = 0.26). Post hoc testing indicated a significant difference on Day 1 (* *p* = 0.0117; Cohen’s *d* = 1.05). Data are presented as mean ± SEM.

Across the cohort, HA concentrations did not change significantly over the 7-day period (*p* = 0.082; partial η^2^ = 0.11), although post hoc comparisons indicated that Day 7 values were higher than several earlier time points. Significant interindividual variability was observed (*p* < 0.0001), indicating maternal differences contributed more to overall variance than acute dietary fluctuations. Median HA concentrations ranged from 89 to 128 ng/mL, while mean values ranged from 108 to 167 ng/mL (Figure 2A).

When stratified by BMI, mothers in the OB group had significantly higher mean HA concentrations than those in the NW group (151.9 vs. 96.5 ng/mL; *p* = 0.0396; Cohen’s *d* = 1.19; Figure 2B). Two-way repeated measures ANOVA confirmed a main effect of BMI group (*p* = 0.028; partial η^2^ = 0.26), whereas neither time (*p* = 0.0820; partial η^2^ = 0.11) nor BMI × time interaction (*p* = 0.196; partial η^2^ = 0.07) were significant. Post hoc analysis showed significantly higher HA levels in the OB group on Day 1 (*p* = 0.0117; Cohen’s *d* = 1.05), with nonsignificant trends toward higher concentrations on Days 2 and 4 (*p* = 0.0549; Cohen’s *d* = 0.78 and 0.71, respectively). Within-group analyses show no consistent temporal pattern, apart from a significant difference between Day 1 and Day 6 in the NW group (*p* = 0.0072).

## 4. Discussion

This exploratory study demonstrated that acute dietary intake did not alter milk HA concentrations during the postprandial period, either in the cohort as a whole or when stratified by BMI. In contrast, across the seven-day temporal stability protocol, a consistent difference emerged, with mothers in the OB group exhibiting higher HA concentrations than the NW group. These findings suggest that acute dietary factors exert minimal influence on milk HA composition, whereas longer-term metabolic or behavioral factors associated with obesity could potentially contribute to sustained elevations in HA levels.

The observed week-long differences may reflect variations in habitual diet and energy balance. Because sampling began on Monday, it is plausible that weekend dietary patterns, typically characterized by higher total caloric intake, contributed to the higher HA concentrations noted later in the week, particularly among mothers with obesity [27,28,29]. This interpretation aligns with our observations, in which HA levels were higher early in the week during collection (day 1 and 2) in OB compared to NW group, converged midweek, and diverged again toward the weekend (day 7). However, in the absence of detailed dietary data, these explanations remain speculative. Future studies incorporating comprehensive dietary and behavioral assessments will be required to determine whether habitual intake patterns contribute to temporal variation in milk HA.

Several obesity-associated mechanisms could contribute to higher HA concentrations in HM. Chronic low-grade inflammation (e.g., elevated tumor necrosis factor alpha [TNF-α], interleukin [IL]-1β) has been shown to activate nuclear factor kappa beta (NF-κB) and induces HA synthases (HAS1-3) in secretory epithelium [23]. Insulin resistance similarly enhances flux through the hexosamine biosynthetic pathway, expanding the intracellular uridine diphosphate N-acetylglucosamine (UDP-GlcNAc) pool, and promoting HAS2 activity/stability via O-linked-GlcNAc-dependent regulation [24]. Obesity has also been linked to altered HA catabolism (e.g., hyaluronidase [HYAL]1/2, cell migration-inducing hyaluronidase 1/transmembrane protein 2 [CEMIP/TMEM2]) [30,31] and higher systemic HA turnover, which may influence mammary HA levels. As maternal inflammatory and metabolic markers were not measured, the relevance of these pathways to the observed differences cannot be determined. Other obesity associated factors, such as cortisol dysregulation, prolactin signaling, and circadian or sleep disturbances, were not captured in this study and may therefore represent unaccounted contributors to the observed differences in milk HA concentrations [32,33].

The functional significance of increased HM HA on infant health remains to be defined. Experimental studies suggest that HA, particularly with molecular weight of 35 kDa (HA35), promotes epithelial barrier integrity and modulates immune signaling through toll-like receptor 4 (TLR4)/CD44-dependent pathways. In both vitro and in vivo models, HA35 supplementation enhances epithelial repair, reduces inflammation, and protects against mucosal injury. In neonatal models, HA35 maintains intestinal permeability, limits bacterial translocation, and attenuates NEC-like injury [17,20,21]. Similarly, in human intestinal organoids, HA35 counteracts hyperglycemia-induced barrier disruption by preserving zonula occludens-1 (ZO-1) and occludin localization through the HA receptor, layilin [34]. Collectively, these findings support a protective role for HA under metabolic and inflammatory stress. Whether chronically higher HA exposure among infants of mothers with obesity confers similar benefits or elicit adaptive or maladaptive responses is unknown. These findings highlight the need for prospective studies linking HA composition, including molecular weight distribution, to infant intestinal, immune, and metabolic development.

Strengths of this study include the use of standardized collection protocols, repeated daily sampling, and the concurrent evaluation of both temporal and dietary influences on milk HA concentrations. Limitations include the small sample size, focus on early postpartum milk, and absence of detailed maternal dietary data or infant outcome measures. An additional limitation is the lack of molecular weight characterization, which is essential given that HA’s biological activity is size dependent [17,35,36]. Although HM typically contains intermediate- to high-molecular-weight HA [37], the absence of direct measurement in this cohort prevents interpretation of the functional significance of the observed concentration differences.

## 5. Conclusions

Acute dietary intake did not alter HA concentrations over the postprandial period, whereas differences between OB and NW mothers emerged across a seven-day interval. These findings could suggest that milk HA reflects longer-term maternal metabolic or behavioral influences rather than immediate dietary effects. Future studies incorporating detailed dietary assessment, HA molecular weight profiling, and infant outcome measures are needed to clarify the physiological significance of obesity-related differences in milk HA composition and their potential impact on infant development.

## Figures and Tables

**Table 1:** Patient demographics.

Patient Demographics	NW (*n* = 19)	OB (*n* = 16)	*p* Value
Age, y (range)	30.0 ± 3.3 (22–34)	30.9 ± 4.0 (25–38)	0.41
Parity	2.29 ± 0.8	1.92 ± 1.08	0.35
Pre-pregnancy Weight (kg)	59.3 ± 5.9	86.5 ± 19.8	<0.0001
Pre-pregnancy Height (cm)	163 ± 6.3	157 ± 9.8	0.03
Pre-pregnancy BMI (kg/m^2^)	22.1 ± 1.4	34.3 ± 4.6	<0.0001

Values are shown as mean ± standard deviation. NW = normal weight (≤25 kg/m^2^); OB = obese (≥30 kg/m^2^).

## Data Availability

The original contributions presented in this study are included in the article/Supplementary Material. Further inquiries can be directed to the corresponding author.

## Supplementary Materials

The following supporting information can be downloaded at: https://www.mdpi.com/article/10.3390/nu17223560/s1, Table S1: Patient Demographics for Study Two.

## Abbreviations

The following abbreviations are used in this manuscript:

HA	Hyaluronan
SEM	Standard error of mean
BMI	Body mass index
OB	Obese
NW	Normal weight
ANOVA	Analysis of variance
HM	Human milk
HMO	Human milk oligosaccharide
NEC	Necrotizing enterocolitis
OUHSC	University of Oklahoma Health Sciences Center
ELISA	Enzyme-linked immunosorbent assay
TNF-α	Tumor necrosis factor alpha
IL	Interleukin
NF-κB	Nuclear factor kappa beta
HAS	Hyaluronan synthase
UDP-GlcNAc	Uridine diphosphate N-acetylglucosamine
HYAL	Hyaluronidase
CEMIP	Cell migration-inducing hyaluronidase
TMEM2	Transmembrane protein 2
TLR4	Toll-like receptor 4
ZO-1	Zonula occludens 1
