# Supplementary material for: Impact of Diet and Maternal Obesity on Human Milk Hyaluronan
Source: Nutrients. 2025 Nov 14;17(22):3560. doi: 10.3390/nu17223560 (PMC12655382; doi:10.3390/nu17223560)
Supplement: Supplementary file 1 [file nutrients-17-03560-s001.zip › nutrients-3970544-supplementary.pdf]

Supplementary Table S1. Patient Demographics for Study Two

Patient Demographics	NW (n=10)	OB (n=8)	P value
**Age, y (range)**	28.8 ± 3.6 (22–33)	31.2 ± 5.1 (26–38)	0.29
**Parity**	2.22 ± 0.83	1.83 ± 0.98	0.17
**Pre-pregnancy Weight (kg)**	57.1 ± 4.83	82.7 ± 8.9	<0.001
**Pre-pregnancy Height (cm)**	160.7 ± 6.6	158.4 ± 6.2	0.55
**Pre- pregnancy BMI (kg/m^2)**	22.1 ± 1.56	33.0 ± 1.88	<0.001

Values are shown as mean ± standard deviation. NW = normal weight (≤25 kg/m^2); OB = obese (≥30 kg/m^2)
